# Bringing the Tiger Back from the Brink—The Six Percent Solution

**DOI:** 10.1371/journal.pbio.1000485

**Published:** 2010-09-14

**Authors:** Joe Walston, John G. Robinson, Elizabeth L. Bennett, Urs Breitenmoser, Gustavo A. B. da Fonseca, John Goodrich, Melvin Gumal, Luke Hunter, Arlyne Johnson, K. Ullas Karanth, Nigel Leader-Williams, Kathy MacKinnon, Dale Miquelle, Anak Pattanavibool, Colin Poole, Alan Rabinowitz, James L. D. Smith, Emma J. Stokes, Simon N. Stuart, Chanthavy Vongkhamheng, Hariyo Wibisono

**Affiliations:** 1 Wildlife Conservation Society, Bronx, New York, United States of America; 2 IUCN/SSC Cat Specialist Group, University of Bern, Bern, Switzerland; 3 Global Environment Facility, Washington, D.C., United States of America; 4 Wildlife Conservation Society, Kuching, Sarawak, Malaysia; 5 Panthera, New York, New York, United States of America; 6 Wildlife Conservation Society, Vientiane, Lao People's Democratic Republic; 7 University of Cambridge, Cambridge, United Kingdom; 8 The World Bank, Washington, D.C., United States of America; 9 Wildlife Conservation Society, Vladivostok, Primorski Krai, Russia; 10 Wildlife Conservation Society, Laksi, Bangkok, Thailand; 11 University of Minnesota, St. Paul, Minnesota, United States of America; 12 IUCN/SSC, University of Bath, Bath, United Kingdom; 13 Wildlife Conservation Society, Bogor, Indonesia; Imperial College London, United Kingdom

The Tiger Summit, to be hosted by Prime Minister Vladimir Putin in Russia in November 2010—the Chinese Year of the Tiger and the International Year of Biodiversity—promises to be the most significant meeting ever held to discuss the fate of a single non-human species. The Summit will culminate efforts by the Global Tiger Initiative (GTI), launched in 2008 by Robert Zoellick, World Bank President. Leaders of 13 tiger range states, supported by international donors and conservationists attending the summit, are being asked to commit to substantive measures to prevent the unthinkable: extinction of the world's last wild tiger populations.

Wild tiger numbers are at an historic low. There is no evidence of breeding populations of tigers in Cambodia, China, Vietnam, and DPR Korea. Current approaches to tiger conservation are not slowing the decline in tiger numbers [1]–[3], which has continued unabated over the last two decades. While the scale of the challenge is enormous, we submit that the complexity of effective implementation is not: commitments should shift to focus on protecting tigers at spatially well-defined priority sites, supported by proven best practices of law enforcement, wildlife management, and scientific monitoring. Conflict with local people needs to be mitigated. We argue that such a shift in emphasis would reverse the decline of wild tigers and do so in a rapid and cost-efficient manner.

## The Decline of the Tiger

Despite a long history of concern for wild tigers, both their range and total number have collapsed: fewer than 3,500 animals now live in the wild, occupying less than 7% of their historical range [4]. Of these, approximately 1,000 are likely to be breeding females [5].

In most countries, overhunting has been the driver of the decline in tigers and their prey [6],[7]. Additionally, loss and fragmentation of habitat was locally important [8]. Nevertheless, beginning in the early 1970s, conservation initiatives helped establish a large number of tiger reserves, particularly in India, Nepal, and, to a lesser extent, in Thailand, Indonesia, and Russia. Probably the most successful of these, at least initially, was Project Tiger in India, which was launched in 1972 with the political support of Prime Minister Indira Ghandi. The apparent success of these reserves prompted, in the 1990s, many conservationists [4],[9],[10] (including some of the co-authors of this report) to shift their focus to a landscape approach, which sought to conserve tigers well beyond protected areas, so as to maintain the genetic and demographic viability of populations of this low-density, wide-ranging species. Conservation investments subsequently increased, but the array of activities was complex, less directly related to tigers, and spread thinly across large landscapes [11]. With hindsight, it also became clear that protection and management of many reserves remained inadequate (the extirpation of tigers in the Indian tiger reserves of Sariska, reported in 2004, and Panna, reported in 2010, is illustrative) and this, coupled with an increased demand for tiger parts [12], meant that poaching of tigers and prey decimated populations across Asia, both inside and outside reserves.

## Protecting Source Sites

**Figure 1:**
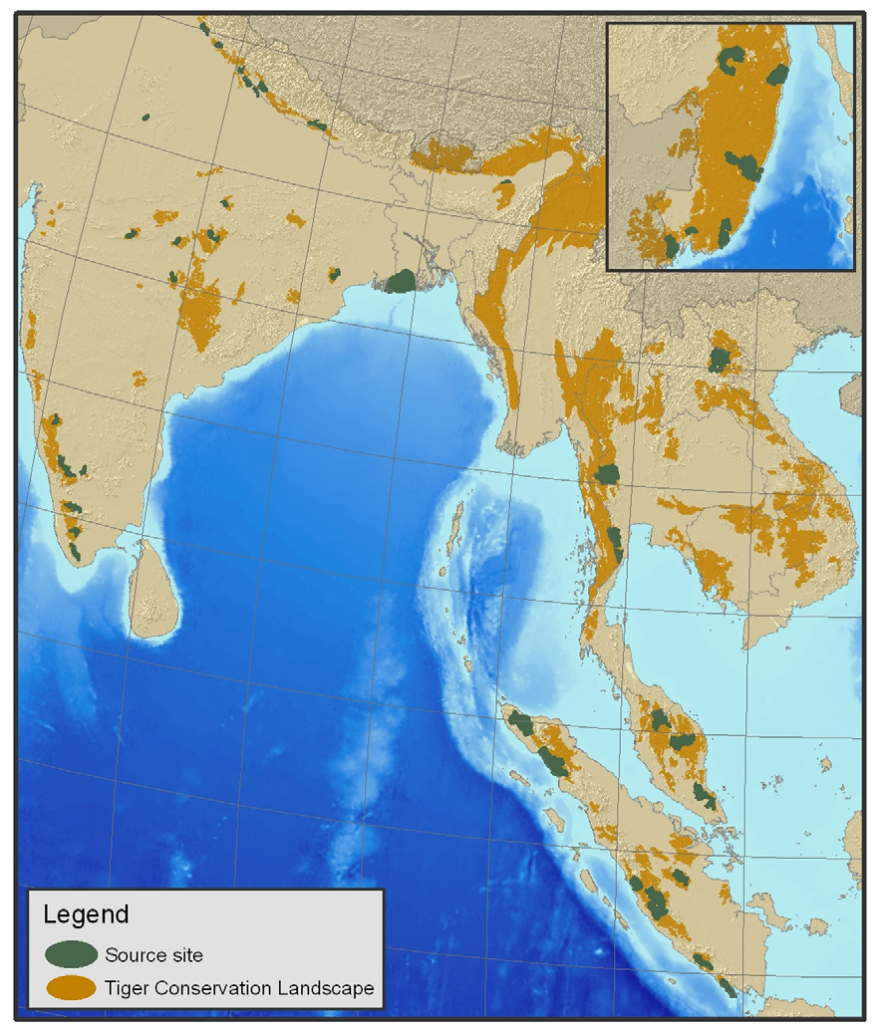
Location of 42 source sites, embedded within the larger tiger conservation landscapes (TCLs), areas that encompass the ecological habitats suitable for tigers.

While approximately 1.5 million square kilometers of suitable habitat still remain in Asia ([9], Figure 1), tigers today are distributed heterogeneously [7],[13] and, except in the Russian Far East, are now restricted to small pockets, mostly in protected areas. The recent analysis ([13], Table S1) identified 42 “source sites,” so termed because these areas contain concentrations of tigers that have the potential to repopulate larger landscapes. Source sites were defined as having the potential to maintain >25 breeding females, being embedded in a larger landscape with the potential to contain >50 breeding females, having an existing conservation infrastructure, and having a legal mandate for protection (Text S1). These sites contain the majority of the world's remaining tigers.

Strategies to save the tiger must focus first and foremost on protecting these remaining concentrations of tigers. These 42 sites contain almost 70% of all remaining wild tigers ([13], Table S1) so have a disproportionate importance to the survival and recovery of the species. Nevertheless, collectively they cover <100,000 km^2^, which is less than 0.5% of their historical range and just 6% of even their current distribution. If Russia is excluded from the analysis, 74% of the world's remaining tigers live in less than 4.5% of current tiger range. Therefore, protecting source sites offers the most pragmatic and efficient opportunity to conserve most of the world's remaining wild tigers.

Source sites are not evenly distributed across the tigers' range (Figure 1). Most are in India (18), Sumatra (eight) and the Russian Far East (six). Based on available data, no source site was identified in Cambodia, China, DPR Korea, or Vietnam [13]. Surveys in Bhutan and Myanmar have thus been too limited for their status to be assessed. Nevertheless, potential source sites in some of these countries warrant further investigation. Even source sites, however, have depressed tiger populations. Only five, all of which are in India, maintain tiger populations close (>80%) to their estimated carrying capacity [13]. Thus, the recovery of populations in source sites alone would result in a 70% increase in the world's tiger population.

While recognizing that the long-term goal is to conserve an Asia-wide network of large, tiger-permeable landscapes, the immediate priority must be to ensure that the last remaining breeding populations are protected and continuously monitored. Without such protection, all other efforts are bound to fail. The similarly dramatic decline in African rhinoceros in the 1980s provides useful lessons on how best to respond to a decline in a species of high commercial value. Where conservation efforts were geographically diffuse, the cost–risk ratio greatly favored the illegal hunter [14]. Only where protection efforts either were focused on small- to medium-sized areas (e.g., Kenya's rhino sanctuaries), or were well financed (e.g., Kruger National Park), did rhinos persist [15]. While tigers have larger spatial requirements than rhinos, the challenge is the same.

Actively protecting tigers at source sites is feasible and pragmatic, and has been demonstrably successful in many reserves across India between 1974 and 1986 [16]. The Malenad-Mysore tiger landscape currently maintains >220 adult tigers, one of the greatest concentrations in the world, mainly due to intensive protection of its source sites such as Nagarahole National Park, where tiger numbers have increased by 400% after protection began in the early 1970s [17],[18], and has now maintained a high density for 30 years ([19], unpublished data). Across India, tiger abundance is strongly correlated with prey density [20] and both depend on strict controls on hunting. The Tigers Forever program [21] has supported governmental protection effort, aided by MIST (Management Information SysTem) law enforcement monitoring [22], in Thailand, Lao PDR, and Malaysia, and hunting has been reduced and tiger populations stabilized. However, these results require greater levels of law enforcement, surveillance, and monitoring than typically is found in national protected areas. In the Russian Far East, traditionally a stronghold for tigers, annual monitoring detected a dramatic decline in tiger numbers over the last five years, which was associated with a decline in enforcement [23],[24]. Recent declines in tiger numbers in the once thriving source sites in Nepal were also associated with reduced emphasis on protection [25].

## The Cost of Protection

We assessed the costs of protecting source sites, including increased law enforcement, biological and law enforcement monitoring, and where appropriate, community engagement, informant networks, and trade monitoring. Costs were sourced, where possible, from those responsible for managing source sites such as protected area authorities, supplemented by published national government figures. Included costs were limited to those supporting the core activities of protection and monitoring of source sites. These include law enforcement, law enforcement monitoring, general management, and the monitoring of tigers and their prey. One-time conservation infrastructure development, and costs related to the relocation of communities within source sites, were not included in the analysis (Text S2).

Protecting source sites is financially attainable. Our analysis [13] estimates the average cost of protecting and monitoring tigers effectively at all 42 source sites at $82 million per year or $930/km^2^ per year, within the range of effective protected area costs in general (from $130 to >$5,000/square kilometer/year for densely settled regions in Asia) [26]. More than half of these funds ($47 million, almost US$500/km^2^) is already being committed by range-state governments and, to a far lesser extent, international donors and NGOs. However, much of the total governmental financial commitment comes from and is spent in India. When India is excluded from the analysis, the average current commitment drops to US$365/km^2^ per year. This leaves an overall shortfall of US$35 million a year for all source sites.

## A Pragmatic Strategy

While protecting source sites is essential to reverse tiger declines, this is but one element of a long-term recovery strategy. For wide-ranging, low-density species like the tiger, conservation planning at the landscape level is necessary, landscapes need to remain permeable to tiger movements, and source sites have to remain embedded in those larger landscapes. This will require strict limits on habitat conversion and infrastructure development. In addition, conservation efforts need to target the illegal trade, as site-based protection will be increasingly costly if the global demand for tiger products is not curtailed [27],[28]. All of this will require concerted, orchestrated and politically bold commitments by range-state governments, supported by the general public and the international community, and sustained over decades.

However, with so few wild tigers remaining, almost entirely clustered in a few small areas, the most immediate need is to protect populations in the remaining source sites. For financially valuable species like the tiger, intensive protection is paramount, and the success of such protection has been demonstrated. Commitments made at the Russian Summit must refocus on the protection of source sites—a strategy that is financially realistic, politically feasible, and will deliver the greatest return on conservation investments. Only when we are able to stop the slide in tiger numbers at source sites will we be successful at managing tigers across the wider landscape.

## Supporting Information

Table S1 **Source sites listed by country.**
(0.07 MB XLS)

Text S1 **Definition of source sites.**
(0.08 MB DOC)

Text S2 **Estimating financial costs for effective protection and monitoring at source sites, and present expenditures.**
(0.06 MB DOC)
