# Supplementary material for: Bringing the Tiger Back from the Brink—The Six Percent Solution
Source: PLoS Biol. 2010 Sep 14;8(9):e1000485. doi: 10.1371/journal.pbio.1000485 (PMC2939024; doi:10.1371/journal.pbio.1000485)
Supplement: Text S1 — Definition of source sites. (0.08 MB DOC) [file pbio.1000485.s002.doc]

**Definition of Source Sites**

The definition of source sites is based on Karanth et al. [1], with some amendments, as those areas embedded within larger landscapes with ‘tiger-permeable habitats’ where tigers are likely to be reproducing above replacement levels and therefore have the potential to repopulate surrounding landscapes. If tigers were lost from sources any natural re-population of larger landscapes would be rendered unlikely.

We define a tiger source site as having the following characteristics:

1. Higher densities of tigers than in the overall landscape within which it is embedded
2. Some evidence of current tiger reproduction
3. Has the potential to maintain a demographically viable cluster of >25 breeding females [2], alone or combined with other connected source sites in the same landscape. The number of 25 breeding females accords with published sources [2,3]. Jhala et al. [4] in the review of sites in India considered 20 breeding females as adequate, while Chapron et al. [5] reviewing Russian tiger populations, considered >80 females as the minimum requisite number for a landscape including several possible source populations.
4. Is embedded within a larger tiger-permeable landscape which has the overall potential to maintain > 50 breeding females
5. A genuine government/social commitment to preventing further human in-migration and/or infrastructure development
6. Existing wildlife protection capacity or at least a political commitments to establish such capacity in the very near future
7. A legal framework in place or being developed for the prevention of poaching or hunting of tigers and their prey

**References**

1. Karanth KU, Goodrich, JM, Vaidyanathan S, Reddy GV (2010) Landscape scale, ecology-based management of wild tiger populations. Washington, D.C.: Global Tiger Initiative, World Bank, and Wildlife Conservation Society.

2. Karanth KU, Stith BM (1999) Prey depletion as a critical determinant of tiger population viability. In: Seidensticker J, Christie S, Jackson P, editors. Riding the Tiger: Tiger conservation in human-dominated landscapes. Cambridge: Cambridge University Press. pp. 100-113.

3. Karanth KU, Nichols JD, Kumar NS, Link WA, Hines JE (2004) Tigers and their prey: Predicting carnivore densities from prey abundance. Proc Natl Acad Sci U S A 101: 4854-4858.

4. Jhala YV, Gopal R, Quereshi Q (2008) Status of tigers, co-predators and prey in India. New Delhi and Dehradun, India: National Tiger Conservation Authority and Wildlife Insitute of India. 152 p.

5. Chapron G, Miquelle DG, Lambert A, Goodrich JM, Legrandre S, et al. (2008) The impact on tigers of poaching versus prey depletion. J Appl Ecol 45: 1667-1674.
