# Supplementary material for: Bringing the Tiger Back from the Brink—The Six Percent Solution
Source: PLoS Biol. 2010 Sep 14;8(9):e1000485. doi: 10.1371/journal.pbio.1000485 (PMC2939024; doi:10.1371/journal.pbio.1000485)
Supplement: Text S2 — Estimating financial costs for effective protection and monitoring at source sites, and present expenditures. (0.06 MB DOC) [file pbio.1000485.s003.doc]

**Estimating Financial Costs for Effective Protection and Monitoring at Source Sites, and Present Expenditures.**

Costs of source site protection were derived from extensive interviews with source site managers and national government agency personnel. Wherever possible, actual expenditures were sourced from those people on the ground responsible for management of the source sites, and agencies directly supporting that management. Official government figures were used where available, though occasionally these figures differed from the funds that the site-based managers were actually able to access.

The actual expenditures included in the analysis were limited to core management and protection activities at the source site and those of the relevant government agency, typically that responsible for parks and protected areas. Activities typically included law enforcement, law enforcement monitoring, general management, and the monitoring of tigers and their prey.

To estimate the additional costs to effectively protect and monitor source sites, managers were encouraged to identify additional activities they consider essential, but were asked to be realistic. Additional activities were restricted to those directly linked to tiger protection. At a number of sites, these activities included community engagement, informant networks, and monitoring of trade routes and local restaurants. Occasionally costs were challenged and removed where they are deemed excessive or sufficiently unrelated to tigers.

The cost of additional or new activities in areas, such as adding essential law enforcement patrols where they are needed but are currently lacking, were adjusted to account for hunting pressure and logistical difficulty of patrolling. These factors influence the nature and expense of activities, and financial estimates were modified accordingly.

Costs relating to the relocation of communities within source sites were not included in this analysis. These costs could not be standardized as the activity is politically and socially complex, and detailed cost estimates were not available for many sites. Voluntary resettlement of families out of critical tiger habitats have in some cases yielded positive results for both communities and wildlife [1], but not always. One-time investments, such as the creation of conservation infrastructure, were also not included.

**References**

1. Karanth KK (2007) Making resettlement work: The case of India’s Bhadra Wildlife Sanctuary. Biol Cons 139: 315-324.
